# The effects of NMDA receptor blockade on TMS-evoked EEG potentials from prefrontal and parietal cortex

**DOI:** 10.1038/s41598-020-59911-6

**Published:** 2020-02-21

**Authors:** Nigel C. Rogasch, Carl Zipser, Ghazaleh Darmani, Tuomas P. Mutanen, Mana Biabani, Christoph Zrenner, Debora Desideri, Paolo Belardinelli, Florian Müller-Dahlhaus, Ulf Ziemann

**Affiliations:** 1 Brain, Mind and Society Research Hub, School of Psychological Sciences and Turner Institute for Brain and Mental Health, Monash University Melbourne, Australia; 2 Hopwood Centre for Neurobiology, Lifelong Health Theme, South Australian Health and Medical Research Institute (SAHMRI), Adelaide, Australia; 3 Discipline of Psychiatry, Adelaide Medical School, University of Adelaide, Adelaide, Australia; 4 Department of Neurology and Stroke, and Hertie Institute for Clinical Brain Research, Eberhard Karls University of Tübingen, Tübingen, Germany; 5 Centre for Cognitive Neuroimaging, Institute of Neuroscience and Psychology, University of Glasgow, Glasgow, United Kingdom; 6 Department of Psychiatry and Psychotherapy, University Medical Center Mainz, Mainz, Germany

**Keywords:** Neuroscience, Neurophysiology

## Abstract

Measuring the brain’s response to transcranial magnetic stimulation (TMS) with electroencephalography (EEG) offers unique insights into the cortical circuits activated following stimulation, particularly in non-motor regions where less is known about TMS physiology. However, the mechanisms underlying TMS-evoked EEG potentials (TEPs) remain largely unknown. We assessed TEP sensitivity to changes in excitatory neurotransmission mediated by n-methyl-d-aspartate (NMDA) receptors following stimulation of non-motor regions. In fourteen male volunteers, resting EEG and TEPs from prefrontal (PFC) and parietal (PAR) cortex were measured before and after administration of either dextromethorphan (NMDA receptor antagonist) or placebo across two sessions in a double-blinded pseudo-randomised crossover design. At baseline, there were amplitude differences between PFC and PAR TEPs across a wide time range (15–250 ms), however the signals were correlated after ~80 ms, suggesting early peaks reflect site-specific activity, whereas late peaks reflect activity patterns less dependent on the stimulated sites. Early TEP peaks were not reliably altered following dextromethorphan compared to placebo, although findings were less clear for later peaks, and low frequency resting oscillations were reduced in power. Our findings suggest that early TEP peaks (<80 ms) from PFC and PAR reflect stimulation site specific activity that is largely insensitive to changes in NMDA receptor-mediated neurotransmission.

## Introduction

Transcranial magnetic stimulation (TMS) is a brain stimulation method capable of non-invasively activating cortical neurons across the scalp in humans via electromagnetic induction^1^. A single TMS pulse evokes a series of time-locked peaks and troughs in electroencephalographic (EEG) recordings of brain activity^2^, which are commonly known as TMS-evoked EEG potentials (TEPs). TEPs are reliable within and between sessions^3–5^, are sensitive to changes in TMS parameters such as intensity^4^ and pulse shape^6^, and differ depending on the cortical site stimulated^4,7^. In addition, TEPs are sensitive to changes in cortical properties resulting from differing brain states, plasticity-inducing brain stimulation paradigms, and brain disorders^8^. As such, TMS-EEG is emerging as a powerful method for investigating cortical dynamics in health and disease.

Despite the recent uptake of TMS-EEG within the brain stimulation field, it remains largely unclear what physiological properties underlie the size, shape and distribution of TEPs, thereby limiting their interpretability. Current hypotheses suggest that TEPs primarily reflect fluctuations in cortical excitability resulting from excitatory and inhibitory neurotransmission at the site of stimulation, as well as the propagation of activation through cortical networks following TMS^8^. However, several recent studies have shown that residual auditory and somatosensory activity resulting from the TMS pulse also contributes to TEPs under certain circumstances despite experimental measures designed to minimise sensory inputs such as auditory masking and foam padding^9,10^. Such findings highlight the need for careful experimental set-up^11^ and control conditions^12^ in TMS-EEG studies. In support of the excitation/inhibition hypothesis, pharmacological agonists of inhibitory neurotransmission mediated by fast activating γ-aminobutyric acid (GABA)-A receptors given at sub-anaesthetic doses increase the amplitude of early TEPs (e.g. N45) following motor cortex stimulation^13,14^, and reduce the propagation of activity following premotor and parietal cortex stimulation at anaesthetic doses^15^. Agonists of slow acting GABA-B receptors at sub-anaesthetic doses, on the other hand, increase the amplitude of latter peaks (e.g. N100) following motor cortex stimulation^13^. Although evidence for the sensitivity of single-pulse TEPs to inhibitory neurotransmission is growing, the effect of excitatory neurotransmission on TEPs is less clear. Several studies have linked early motor TEPs between 15–40 ms after TMS with fluctuations in cortical excitability measured via motor-evoked potentials^16–18^, however this association has been challenged^19^. Furthermore, TEPs following single-pulse TMS of premotor or parietal cortex are largely unaffected following anaesthetic doses of ketamine, an n-methyl-d-aspartate (NMDA) receptor antagonist^20^. To date, no studies have assessed the sensitivity of single-pulse TEPs to changes in NMDA receptor-mediated neurotransmission while individuals are conscious.

The primary aim of this study was to investigate the contribution of NMDA receptor-mediated neurotransmission to the generation and propagation of TEPs following single-pulse TMS in conscious, healthy adults. We measured TEPs following prefrontal (PFC) and parietal (PAR) cortex stimulation before and after a sub-anaesthetic dose of dextromethorphan, an NMDA receptor antagonist, or a placebo in a double-blinded pseudo-randomized crossover design. We hypothesised that early (15–40 ms) TEPs would be reduced following dextromethorphan, but not placebo. Given that recent studies have suggested some of the TMS-EEG signal may reflect TMS-evoked sensory activity common across stimulation sites^9,10^, we compared the differences and similarities between TEPs following stimulation of the different sites to determine which aspects of the TEPs were site specific. As there is currently no consensus on the best way to process TMS-EEG data^21^, we also assessed the impact of different cleaning pipelines on the study outcomes. Finally, we compared the effect of dextromethorphan on resting-state oscillations, which are typically reduced in power within low-frequency and increased in high-frequency bands following NMDA receptor antagonists^22^.

## Materials and Methods

### Participants

Fifteen right-handed male participants were recruited for the study. Data from one participant was removed due to a fault in the TMS noise-masking in one condition, leaving a total of fourteen participants (mean age ± S.D. = 28.7 ± 5 years, range = 21–39 years). Female participants were excluded due to the possible confounding effects of the menstrual cycle on TMS-evoked cortical excitability^23^. Prior to enrolment, the medical history was taken in all candidates, including a neurological and general physical examination to rule out any neurological, psychiatric or medical conditions. Then, the candidates were screened for contraindications to TMS^24^. Exclusion criteria included: presence or history of neurological or psychiatric disease, current use of central nervous system active drugs, abuse of recreational drugs including nicotine or alcohol, or contraindications to dextromethorphan. The experimental procedures were approved by the local ethics committee of the Eberhard-Karls-University Medical Faculty, Tübingen (protocol 526/2014BO1), and all participants provided signed, informed consent in accordance with the latest version of the Declaration of Helsinki.

### Experimental design

Participants underwent a pseudo-randomised, placebo-controlled, double-blind cross-over experiment to assess the effects of dextromethorphan on TEPs resulting from PFC and PAR stimulation. Dextromethorphan is a non-competitive antagonist of the glutamatergic NMDA receptor, but also interacts with serotonin transporters, sigma-1 receptors, and α3β4 nicotinic acetylcholine receptors^25^. Prior to the experimental sessions, all participants underwent a T1-weighted magnetic resonance imaging (MRI) scan of their brain for use in TMS neuronavigation and EEG electrode position digitisation. Participants then attended two experimental sessions at least one week apart. During testing, participants were seated comfortably in a chair with hands resting on a pillow in their lap. Baseline measures included: systolic and diastolic blood pressure, resting motor threshold (RMT), two 4 min periods of resting EEG (eyes open and closed; measured to assess the impact of dextromethorphan on resting oscillations), and TEPs following stimulation of PFC and PAR. Following baseline measures, participants ingested either 120 mg of dextromethorphan (dosage based on previous TMS studies showing significant pharmacological effects^26,27^) or placebo (session order pseudorandomised across subjects). After 60 min, blood pressure, resting EEG, and TEP measures were repeated. 60 min was chosen based on dextromethorphan pharmacokinetics, with blood plasma levels peaking ~60–120 min after drug ingestion^28^. Blood pressure was measured again at the end of the experimental session.

### MRI

A T1-weighted anatomical MRI scan of the brain was acquired from each subject using a 3 T MRI scanner (MAGNETOM^®^ Prisma^fit^, syngo MR D13D, Siemens Healthcare GmbH. Voxel size = 1 × 1 × 1 mm^3^; FoV read = 250, FoV phase = 93.8%, TR = 2300 ms, TE = 4.18 ms, FA = 9.0°).

### EEG

EEG was recorded from 62 TMS-compatible, c-ring slit electrodes (EASYCAP, Germany) using a TMS-compatible EEG amplifier (BrainAmp DC, BrainProducts GmbH, Germany). Data from all channels were referenced to the FCz electrode online with the AFz electrode serving as the common ground. EEG signals were digitised at 5 kHz (filtering: DC-1000 Hz) and EEG electrode impedance was kept below 5 kΩ throughout the experiment. Electrode positions were digitised to each individual’s T1-weighted MR image using a frameless stereotaxic neuronavigation system (TMS Navigator, Localite GmbH, Germany). During eyes open resting EEG, participants were asked to look at a fixation cross and blink as normal. During eyes closed, participants were asked to close their eyes and avoid going to sleep.

### TMS

For TEPs, two sites were stimulated using monophasic TMS pulses (Magstim company, UK): left superior frontal gyrus (PFC; MNI coordinates: −20, 35, 55) and left superior parietal lobule (PAR; −20, −65, 65). We deliberately chose sites close to the midline to minimise TMS activation of scalp/facial muscles^29,30^. Monophasic TMS pulses (current flow = latero-medial in brain to run perpendicular to gyrus) were given through a figure-of-eight coil (external diameter = 90 mm) connected to a Magstim 200^2^ unit (Magstim company, UK). The TMS coil position was determined and monitored throughout the experiment using frameless stereotaxic neuronavigation co-localised to individual T1-weighted MR scans (TMS Navigator, Localite GmbH, Germany). Coil angle was positioned so that the coil handle ran perpendicular to the underlying gyrus with the handle pointing laterally. As there are currently no standardised methods for determining TMS intensity in non-motor regions, TMS intensity was set to 100% of resting motor threshold (RMT) for each site. At the beginning of each experiment, the motor hotspot for the right first dorsal interosseus (FDI) muscle was determined over left primary motor cortex as the site where slightly suprathreshold TMS pulses consistently elicited motor-evoked potentials (MEPs) in the right FDI. Electromyography was recorded using Ag-AgCl electrodes placed in a belly tendon montage over the target muscle (filter: 20–2000 Hz; sampling rate: 5 kHz). RMT (in % maximum stimulator output; MSO) was then determined as the minimum intensity to evoke at least 5 of 10 MEPs > 50 μV peak-to-peak amplitude. For the experimental conditions, 150 TMS pulses were delivered at a rate of 0.2 Hz ± 25% jitter for each site and the order of sites was randomised at each measurement point. Participants were asked to look at a fixation cross during stimulation and blink as normal. Muscle activity and excessive eye movement were monitored by an experimenter throughout the session and fed back to the participant via a tap on the shoulder if too high.

### EEG analyses

Analyses were performed in MATLAB r2017a (MathWorks Inc.) using EEGLAB (v14.1.1)^31^, TESA (v0.1.0)^21^, FieldTrip (v20170815)^32^, Brainstorm (v20180108)^33^, and FreeSurfer (v5.3)^34,35^ toolboxes, and custom code. All custom code is available at: (https://github.com/nigelrogasch/DXM_TMS-EEG_paper).

#### TMS-EEG

As we were interested in early TEP peaks, we developed a novel TMS-EEG cleaning pipeline including two analysis methods designed to recover early TMS-evoked activity (<45 ms) from TMS-related artifacts; the source-estimate-utilizing noise-discarding (SOUND)^36^ algorithm and signal-space projection source-informed reconstruction (SSP-SIR)^37^. For each site and time point, the data were epoched around the TMS pulse (−1500 to 1500 ms), data from −2 to 6 ms around the TMS pulse were removed and replaced with baseline data, and the average between −1000 to 1000 ms was subtracted from each epoch. Line noise was removed by fitting and subtracting a 50 Hz sine wave from the EEG time courses using linear regression, and bad channels were identified using a data-driven Wiener-estimation approach and removed^36^. Data were then submitted to independent component analysis (extended infomax) and components representing TMS-evoked muscle artifacts or blinks were detected using the TESA *compselect* function (default settings) and manually checked before being removed^21^. A high-pass filter (1 Hz, zero-phase Butterworth filter, order = 4) was applied and trials containing excessive muscle activity or movement were removed. SOUND was then applied to suppress TMS-evoked decay and other noise-related signals^36^. During this procedure, missing electrodes were replaced with the SOUND estimates and the data were re-referenced to average. A second round of ICA was applied, and components representing ongoing muscle activity were detected using the TESA *compselect* function (default settings) and manually checked before being removed, with special care taken not to remove components representing a mix of neural and artifactual signal. SSP-SIR was then used to suppress any remaining early TMS-related artifacts as required^37^. Finally, the data were downsampled (1000 Hz), low-pass filtered (100 Hz, zero-phase Butterworth filter, order = 4), re-epoched to remove possible boundary artifacts (−1000 to 1000 ms), and re-baseline corrected (−500 to −5 ms). See Table S1 for number of trials, channels and components removed.

As there is currently no consensus on the best pipeline for cleaning TMS-EEG data, we re-cleaned the data using a pipeline we have used previously^38^ and repeated the analyses to assess whether the cleaning procedure impacted the outcomes of the study (see supplementary methods for details and Table S2 for number of trials, channels and components removed).

In addition to scalp analysis, we also applied source estimation using two different methods, dipole fitting and minimum-norm estimation (MNE)^39^, to assess which cortical regions most likely explained the EEG scalp data. For the forward model, each individual’s T1 scan was automatically segmented using the FreeSurfer software. After visual inspections and manual corrections, the FreeSurfer output was imported to Brainstorm and the cortical surface was down sampled to 15,000 vertices. Registration between EEG and MRI was then performed by aligning the locations of EEG electrodes with the generated scalp surfaces. The head model was computed using a three-layer symmetric boundary element method via OpenMEEG^40^, with default conductivity values (scalp = 1, skull = 0.0125 and brain = 1). For dipole fitting, each TEP topography measured at each point of time was assumed to be generated by one freely orientating current dipole located somewhere among the cortical vertices. Each of the modelled current dipoles was independently fitted to the TEP topography (least-square fit) and the location of the dipole with the best goodness-of-fit (GOF) was taken as the most likely point of TMS-evoked cortical activity^41^. For MNE, the cortical distributed sources were formed of freely orientating dipoles using the *l*^2^-MNE solution^39^, which was regularised with singular value decomposition using the dimensions corresponding to the 15 largest components^39^.

#### Resting EEG

Eyes open and eyes closed resting EEG were cleaned using identical pipelines. For each condition and time point, data were downsampled (1000 Hz), bandpass (1–100 Hz) and bandstop (48–52 Hz) filtered using a zero-phase Butterworth filter (order = 4), epoched into non-overlapping 2 s segments, and concatenated into a single file for each session containing eyes open and eyes closed data from pre and post drug intake measurement time points. The data were then visually inspected, and segments with excessive muscle or eye activity and noisy channels (e.g. from disconnected electrodes) were removed. Data were then submitted to the FastICA algorithm, and independent components representing blinks, eye movement, muscle activity or electrode noise were detected using the TESA *compselect* function (default settings) and manually checked before being removed. Finally, removed channels were replaced and data were re-referenced to the average of all electrodes, and separated back into individual conditions and time points. To quantify resting oscillations, data from each segment were converted into the frequency domain using a Fourier transform with a single taper Hanning window (linear trends removed; frequency resolution = 1 Hz) and then averaged across segments. See Table S3 for details on number of segments, channels and components removed.

### Statistics

#### TEP comparisons between stimulation sites

To assess differences in TEPs following PFC and PAR stimulation, baseline TEPs were compared between stimulation sites for each condition using cluster-based permutation statistics (cluster threshold: p < 0.05 dependent t-test; cluster alpha < 0.05 two-tailed; randomisation = 5000; time included: 15–250 ms). To assess similarities between stimulation sites, Spearman’s correlations were performed on TEP amplitudes across electrodes (scalp) and vertices (source) for each time point, converted to z scores, and compared with baseline measures using Mann-Whitney U tests.

#### Effects of dextromethorphan on TEPs and resting oscillations

Cluster-based permutation statistics were used to compare changes in TEP amplitude and resting oscillations across time following dextromethorphan and placebo administration, and between conditions by comparing post values subtracted from pre values (cluster threshold: p < 0.05 dependent t-test; cluster alpha < 0.05 two-tailed; randomisation = 5000). TEP analyses included a broad time range (i.e. no *a priori* assumptions about peak times; 15–250 ms), and at six peaks evident following PFC and PAR stimulation (cluster alpha < 0.008; Bonferroni corrected to control the false-discovery rate testing over six peaks). For cluster-based permutation test on individual peaks, peak times were selected separately for each stimulation site by averaging baseline TEPs from each condition, and using a peak detection algorithm on the global mean field average. Data from the peaks were taken as the average of the peak ±5 ms (peaks at <100 ms latency to TMS) or ±15 ms (peaks at >100 ms latency to TMS). For PFC stimulation, two early peaks were not identifiable in the global mean field average, and were taken from the Fz electrode instead. Data from TEP peaks were also compared using Bayes Factor (BF) analysis to assess evidence for the null hypothesis that changes in peak amplitudes did not differ following dextromethorphan or placebo (JASP v0.8.1.2; Cauchy prior = 0.07; BF_01_ > 3 taken as moderate evidence). For Bayes Factor analysis, data from the six highest amplitude electrodes were averaged for each peak and post values subtracted from the pre values to create a single change score for each condition. For resting oscillations, data were averaged into five canonical oscillation bands prior to cluster-based analysis: delta (1–3 Hz); theta (4–7 Hz); alpha (8–12 Hz); beta (13–29 Hz); and gamma (30–45 Hz) (cluster alpha < 0.01; Bonferroni corrected to control the false-discovery rate testing over five bands). Spearman’s correlations were used to assess relationships between changes in resting oscillatory power and TEP peak amplitudes (alpha < 0.008; Bonferroni corrected to control the false-discovery rate testing over six peaks).

## Results

All experimental procedures were generally well tolerated, with several individuals reporting mild dizziness and one individual nausea following dextromethorphan. These side effects did not affect the subjects’ capacity to fully comply with study requirements. There was no difference in RMT at baseline between drug conditions (dextromethorphan = 48.4 ± 8% MSO; placebo = 47.8 ± 8% MSO; p = 0.09, paired sample t-test). Changes in blood pressure did not differ between conditions (supplementary results).

### Baseline TEPs following PFC and PAR stimulation

**Figure 1:**
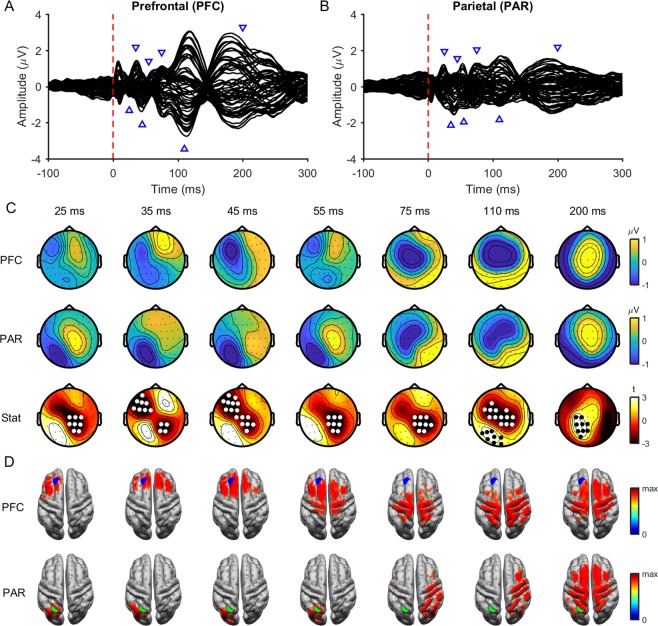
Comparison of baseline TEPs between stimulation sites. Butterfly plots of grand average TEPs across all individuals following prefrontal (PFC; **A**) and parietal (PAR; **B**) cortex stimulation at baseline (averaged across conditions). The red dashed line represents the timing of the TMS pulse and the blue triangles the latencies plotted in (**C,D**). (**C**) Topoplots showing the grand average amplitude of TEPs at different time points following PFC (top row), and PAR stimulation (middle row). The bottom row shows t-statistics comparing the amplitude of PFC and PAR stimulation. White and black dots indicate significant negative and positive clusters (cluster-based permutation tests on 15–250 ms; 2 positive clusters [p = 0.040, 81–142 ms; p = 0.006, 148–250 ms]; 1 negative cluster [p = 0.002, 15–192 ms]). (**D**) Minimum-norm estimate source maps averaged across participants showing peak activity at each time point in C following PFC (top row) and PAR (bottom row) stimulation. Activity has been thresholded to 85% of maximum activity at each time point. The blue dot represents the target for PFC stimulation and the green dot the target for PAR stimulation.

**Figure 2:**
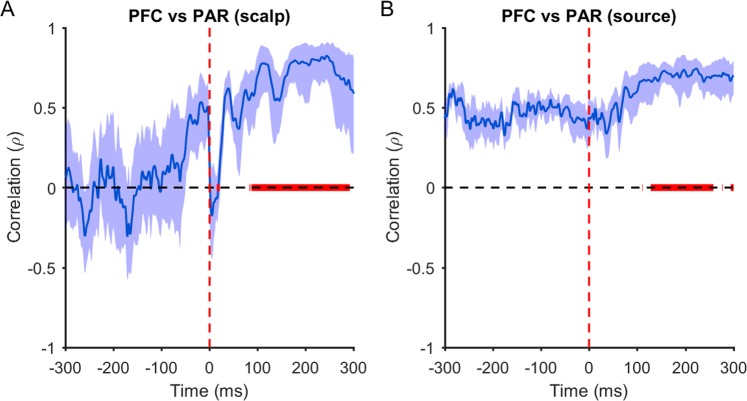
Spatial correlations between prefrontal (PFC) and parietal (PAR) TEPs. Spearman correlations comparing the relationship between PFC and PAR TEPs at the scalp (**A**) and source (**B**, using MNE, cf. Fig. 1D) level for each time point. The thick blue line represents the mean rho values across individuals, and the shaded bars the 95% confidence intervals. The thick red line indicates post stimulation time points where correlations are greater than at equivalent pre stimulation time points (p < 0.05; Mann-Whitney U test). Note that rho values were converted to z for statistics, then back to rho for plotting.

We first assessed the differences and similarities between TEPs following stimulation of different sites. We could not detect any differences in TEP amplitudes between baseline recordings for each site (all p > 0.25) indicating that TEPs are reliable within individuals between sessions, so we averaged across baseline conditions to maximise TEP signal strength. When comparing across a broad time window (15–250 ms), TEPs following PFC stimulation differed in amplitude compared with PAR stimulation across all time points (Fig. 1). Despite the amplitude differences, the spatial distribution of TEPs were highly correlated between stimulation sites after ~83 ms (Fig. 2A), suggesting that later peaks may represent similar underlying cortical sources regardless of the stimulated sites.

To further explore the origin of early and late TEPs, we applied two different source estimation methods: dipole fitting and MNE. For early peaks, the location of the best fitting dipole tended to be closer to the site of stimulation compared to the non-target site (e.g. the PAR when the PFC was stimulated and *vice versa*; Table 1). Note that the difference between the target and non-target dipole location for the early peak following PFC stimulation was not significant using pipeline one, but was significant following pipeline two (see Table S4). In contrast, the dipole locations corresponding to late peaks were closer to the PAR target regardless of stimulation site. For MNE, estimated source distributions were located close to the site of stimulation for early peaks (25–55 ms; Fig. 1D), showed some overlap between stimulation sites for middle peaks (75,110 ms), and were similar for late peaks (200 ms). Similar to the scalp data, MNE spatial distributions were highly correlated between PFC and PAR TEPs from ~129 ms to ~259 ms (Fig. 2B). Taken together, these findings suggest that early TEP peaks reflect neural activation specific to the site of stimulation, whereas late peaks reflect common activation patterns irrespective of the site of stimulation, which differ in amplitude between stimulation sites.

**Table 1:** Distance from TMS target sites to best-fitting dipoles at baseline.

	Distance from target (mm)	Distance from non-target (mm)	Goodness of fit (GoF)	p-value
PFC (15–45 ms)	60 [18–133]	73 [40–103]	0.93 [0.81–0.99]	0.135
PFC (95–125 ms)	80 [24–129]	59 [20–110]	0.88 [0.68–0.99]	0.077
PFC (175–205 ms)	80 [37–123]	**51 [21–122]**	0.84 [0.69–0.99]	0.003
PAR (15–45 ms)	**49 [23–91]**	97 [68–130]	0.93 [0.69–0.99]	4.7 × 10^−5^
PAR (95–125 ms)	**52 [22–94]**	87 [51–110]	0.90 [0.79–0.97]	1.5 × 10^−4^
PAR (175–205 ms)	**44 [19–85]**	82 [42–130]	0.87 [0.72–0.99]	0.001

NB: Values in column 1–3 represent the mean [range]. Bold numbers indicate which site was closest to the best fitting dipole (target vs. non-target; p < 0.05, Mann-Whitney U test). PFC, prefrontal cortex; PAR, parietal cortex.

### Effect of dextromethorphan on TEPs

**Figure 3:**
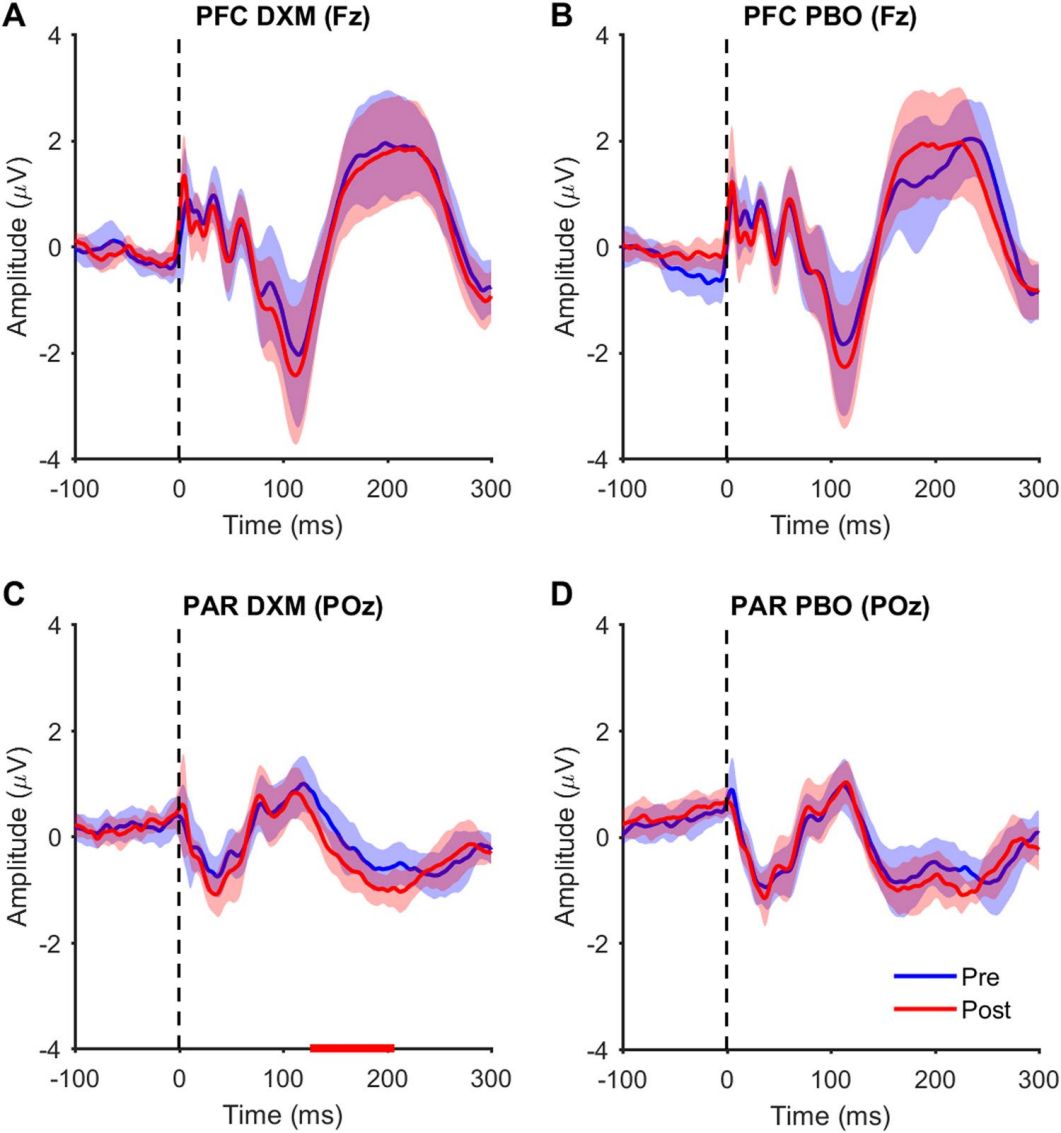
TEPs from single electrodes following dextromethorphan (DXM) and placebo (PBO). (**A,B**) TEPs measured from the Fz electrode following prefrontal cortex (PFC) stimulation pre and post dextromethorphan (DXM) and placebo (PBO) administration. (**C,D**) TEPs measured from the POz electrode following parietal cortex (PAR) stimulation pre and post dextromethorphan and placebo administration. Thick coloured lines represent the group mean and shaded colour lines represent 95% confidence intervals. Red line on x-axis in C represents time period of significant cluster (p < 0.05) between pre and post.

**Figure 4:**
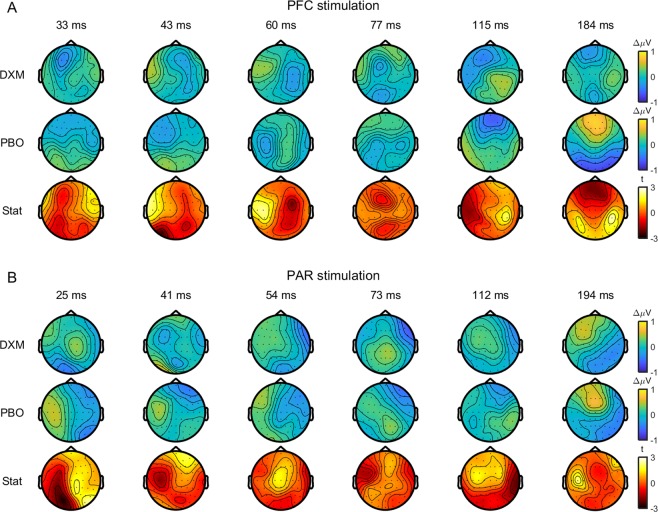
Comparison of changes in TEPs following dextromethorphan (DXM) and placebo (PBO). Topoplots showing changes in TEP amplitude at peak latencies following prefrontal (PFC; **A**) and parietal (PAR; **B**) cortex stimulation after dextromethorphan (top row) and placebo (middle row). Topoplots showing t-statistics (within-subject t-tests) comparing TEP changes between dextromethorphan and placebo are shown on the bottom row. No significant differences were observed between conditions (cluster-based permutation tests).

We next assessed whether dextromethorphan altered TEP amplitudes. We could not find any differences in TEP amplitudes across time following either dextromethorphan or placebo for PFC stimulation (all p > 0.05; Fig. 3A,B), whereas there was a change in PAR TEP amplitude following dextromethorphan (positive cluster, p = 0.006, 126–207 ms; negative cluster, p = 0.0132, 125–201 ms; Figs. 3C and S1), but not following placebo (p > 0.05; Fig. 3D). However, these changes were not replicated when analysing the data with a different cleaning pipeline (p = 0.102; Fig. S2), and we could not find any difference between conditions when directly comparing the change in TEP amplitudes following dextromethorphan and placebo for either stimulation site (all p > 0.05; 15–250 ms), suggesting the changes observed following dextromethorphan with PAR stimulation were not robust. To ensure that the size of later clusters was not biasing the analysis against smaller earlier clusters, we reran the analysis averaging across shorter time windows capturing the main TEP peaks, but could not detect any differences across time or between conditions (all p > 0.05; Bonferroni corrected; Fig. 4). We then ran Bayesian t-tests over ROIs for each peak (determined from baseline data) to assess evidence for the null hypothesis that changes in TEP amplitudes did not differ between conditions. For all comparisons, the BF_01_ was between 1–4, providing weak/moderate evidence that changes in TEP peak amplitude did not differ between dextromethorphan and placebo (Table 2).

**Table 2:** Bayes factors comparing the change in TEP peak amplitude following dextromethorphan (DXM) vs. placebo (PBO).

TEP peaks	DXM vs PBO	
	PFC (BF_01_)	PAR (BF_01_)
33, 25	**3.0**	1.3
43, 41	2.5	**3.7**
60, 54	**3.6**	2.6
77, 73	**3.7**	**3.6**
115, 112	**3.6**	1.9
184, 194	**3.4**	**3.0**

NB: Values in column one represent the mean TEP peak latency for prefrontal (PFC) and parietal (PAR) cortex stimulation respectively. Bold numbers indicate moderate evidence for no difference between conditions.

### Effect of processing pipeline on TEP results

As we used a novel TEP cleaning pipeline, we reran all of the analyses using a more conventional pipeline with two rounds of ICA^21,38^. As with pipeline one, we found differences in amplitude and source localisation of early TEP peaks between stimulation sites, high correlations in scalp topography and source distribution between sites for later peaks, and non-significant effects of dextromethorphan on TEPs following PFC stimulation (Figs. S2–S5; Tables S4 and S5). In contrast to pipeline one, we could not find any evidence for changes in PAR TEP amplitudes following dextromethorphan administration.

### Effect of dextromethorphan on resting oscillations

**Figure 5:**
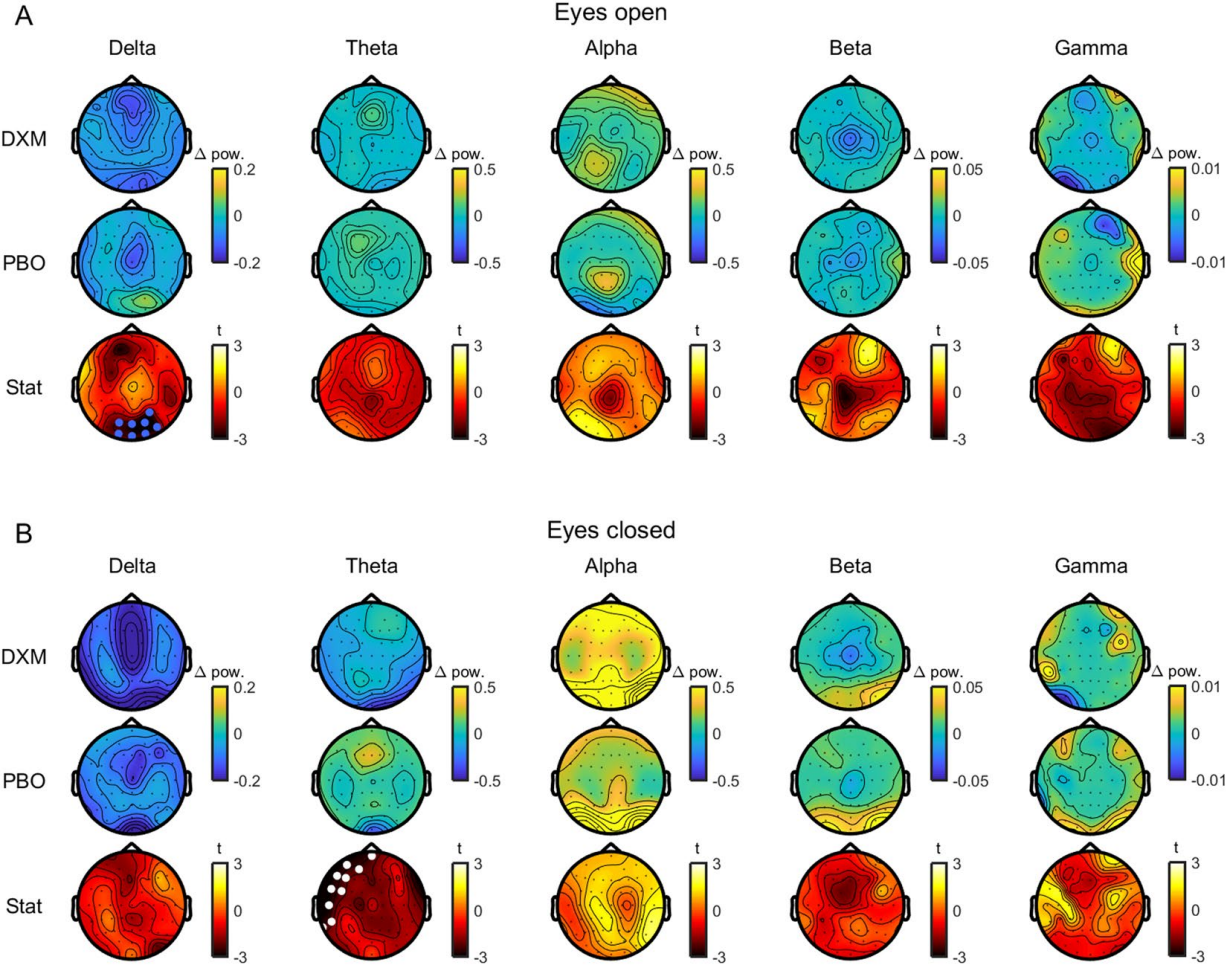
Comparison of changes in resting oscillations following dextromethorphan (DXM) and placebo (PBO). Topoplots showing changes in oscillatory power in different frequency bands during eyes open (**A**) and eyes closed (**B**) resting conditions following dextromethorphan (top row) and placebo (middle row). Topoplots showing t-statistics (within-subject t-tests) comparing power changes between dextromethorphan and placebo are shown on the bottom row. White dots indicate significant clusters with Bonferroni correction and blue dots uncorrected clusters.

As dextromethorphan did not have a robust effect on TEPs, we assessed whether resting oscillations were altered to ensure that the dose of dextromethorphan was sufficient to alter neural activity. We could not detect any differences in resting oscillations at baseline between sessions (all p > 0.05), suggesting that the spatio-spectral profile of oscillations was stable across sessions within individuals. Delta oscillatory power was reduced following dextromethorphan in the eyes open (p = 0.002) and eyes closed (p = 0.009) conditions, whereas beta oscillatory power was reduced following placebo in the eyes closed condition only (p = 2.0 × 10^−4^). When comparing conditions, reductions in delta power tended to be larger following dextromethorphan than placebo for eyes open (p = 0.013; Fig. 5A), although this did not survive correction for multiple comparisons, whereas a reduction in theta power was larger following dextromethorphan than placebo for the eyes closed condition (p = 0.009; Bonferroni-corrected; Fig. 5B). We could not detect differences in oscillatory power changes between dextromethorphan and placebo for any other frequency band (all p > 0.05). Taken together, these findings suggest that dextromethorphan reduces power in low frequency oscillations (delta and theta) during resting states. Finally, we assessed whether changes in resting delta oscillatory power in the eyes-open condition and theta oscillatory power in the eyes-closed condition correlated with changes in TEP peak amplitudes at each site. We reasoned that if the drug did alter TEP peak amplitudes, but this effect was too small to be detected in the group analysis, then these changes should still correlate with changes in resting-state oscillations. We could not find any evidence for a relationship between changes in resting delta oscillatory power (over occipital electrodes) and TEP peak amplitudes following dextromethorphan (all p > 0.13; Spearman’s correlations). In contrast, changes in theta oscillatory power (over left frontal electrodes) were negatively correlated with changes in TEP peak amplitude at 41 (r_s_ = −0.74, p = 0.004), 55 (r_s_ = −0.71, p = 0.006) and 194 ms (r_s_ = −0.82, p = 0.001) following PAR, but not PFC stimulation (all p > 0.08) (Figs. S6 and S7); however, these relationships were not replicated using pipeline two (all p > 0.15; Figs. S8 and S9). Together, these analyses support our findings that TEP peak amplitudes following PFC stimulation are not altered by dextromethorphan, although the evidence is less clear for TEPs following PAR stimulation.

## Discussion

TEPs offer unique insight into the effects of TMS on local cortical circuits and networks, however the precise mechanisms reflected by TEPs remain largely unclear. In the current study, we have shown that early TEPs (<50 ms) are localised to regions close to the site of stimulation, whereas late peaks (>80 ms) showed common activation patterns, independent of the stimulated sites. We also provide weak/moderate evidence that early TEP peaks are not altered by dextromethorphan, although the findings are less clear for later time periods, especially following PAR stimulation. Our findings confirm that TEPs are sensitive to the site of stimulation and provide a deeper understanding of the physiological mechanisms reflected by TEPs elicited by prefrontal and parietal cortex stimulation.

### Dependence of TEPs on stimulation site

Studies directly comparing TEPs following stimulation of different cortical sites have shown both differences and similarities in the local response profile and the cortical networks activated by TMS. For instance, the local oscillatory profile following TMS appears to differ along an anterior-posterior gradient, with frontal sites oscillating at higher frequencies than parietal and occipital sites following stimulation^6^. Furthermore, stimulation of motor cortex results in larger TEPs than non-motor regions^42^, with a unique oscillatory profile^43^. The broader cortical networks activated following TMS also differ depending on the stimulation site, even within stimulation of functionally-related regions^44^.

Despite the differences in TEPs following stimulation of different cortical sites, several studies have reported similarities in TEPs regardless of the target site, especially at periods ~100 ms, and ~200 ms following stimulation^45^. These periods coincide with auditory-evoked potentials resulting from the TMS clicking noise, and bone-conducted sensory responses from coil vibration^46^. To minimise sensory contamination, noise-masking is typically provided during stimulation (e.g. white noise played through headphones) and/or foam is placed under the coil to minimise vibration^47^. Even with such measures, several recent studies have reported that TEPs are highly correlated with control conditions (e.g. TMS of the shoulder or electrical stimulation of the scalp)^9,10,48,49^.

In the current study, we applied auditory masking, and stimulated sites close to the midline to minimise sensation resulting from the activation of scalp muscles with TMS. We found differences in TEP amplitudes following stimulation of PFC and PAR at the scalp level across a broad time range (15–250 ms). However the spatial distribution of the TEPs were highly correlated between sites from ~80 ms onwards. Source estimation using two different methods (dipole fitting and MNE) suggested that the early TEP response (15–55 ms) reflected activity from regions close to the site of stimulation, whereas late TEP responses reflected activity from partially or fully overlapping central regions regardless of stimulation site. These findings most likely suggest that early TEP responses represent TMS-evoked cortical activity from the site of stimulation. We cannot rule out that the early differences in scalp topology between stimulation sites are due to differences in somatosensory input, as we did not include a sensory control condition in this study. However, we think this interpretation is unlikely as tactile stimulation of different scalp and facial sites results in early sensory responses within the contralateral primary somatosensory cortex, which are very similar in latency (peak at ~40 ms) and location when measured using magnetoencephalography (MEG)^50^. In contrast, the early peaks following TMS (25–45 ms) localised to the site of stimulation and were clearly different from one another. Regarding the high correlation in scalp and source topographies of later peaks between stimulation sites, the most likely explanation is that part of the late TEP response reflects indirect activation of the cortex from sensory input, regardless of the efforts to minimise TMS-evoked sensation and audition. Another possibility for explaining similarities in spatial distribution of late TEPs in the present study is that areas of the fronto-parietal network were stimulated potentially leading to common network activation at late time points. Future work comparing the similarities and differences between TMS-evoked and sensory-evoked activity following stimulation of different sites with active and control conditions (e.g. electrical scalp stimulation with a coil click away from the scalp) is required to further disentangle the origin of the various TEP peaks.

### Effects of dextromethorphan on TEPs

Pharmacological studies targeting inhibitory receptors have provided evidence that certain TEP peaks around 45 and 100 ms are sensitive to changes in GABAergic neurotransmission^13,14^, whereas peaks at 30 ms, 45 ms and 180 ms are sensitive to anti-epileptic drugs targeting voltage-gated sodium channels^51,52^. However, the sensitivity of TEPs to changes in excitatory neurotransmission is less clear. Several lines of indirect evidence suggest that early TEP peaks between 15 to 40 ms may reflect excitatory neurotransmission. First, the amplitude of early TEP peaks in motor cortex (N15, P30) correlate with fluctuations in MEP amplitude (which reflect activation of the corticomotoneuronal system)^16^, and show similar changes with TMS intensity^53^, coil angle^17^, and paired pulse paradigms^18^ to MEPs, suggesting that both measures reflect fluctuations in cortical excitability. However, changes in MEPs and early TEPs are not related following continuous theta burst stimulation, questioning the veracity of this relationship^19^. Second, excitatory postsynaptic potentials generated by NMDA receptor activation peak at ~15–40 ms in rodents following electrical stimulation of the neocortex^54,55^, latencies which are similar to early TEP peaks. Collectively, this body of evidence has led to the hypothesis that early TEP peaks may reflect fluctuations in excitatory and inhibitory postsynaptic potentials following TMS mediated by α-amino-3-hydroxy-5-methyl-4-isoxazolepropionic acid (AMPA), NMDA and GABA receptors, although direct evidence for a specific role of NMDA receptors is weak^20^.

We could not find any reliable evidence that changes in early TEP peaks differed following administration of the NMDA receptor antagonist dextromethorphan compared to placebo following stimulation of either site. We did find a difference in parietal TEP amplitude following dextromethorphan administration which was largest between 125–201 ms and a relationship between changes in TEP peaks at 41, 55, and 194 ms following PAR stimulation and changes in theta oscillatory power during eyes-closed resting state. However, these findings did not replicate when using a different cleaning pipeline, and we could not find differences in TEP changes when directly comparing the active and placebo conditions, suggesting the findings were not overly robust. Although our sample was relatively small (n = 14), Bayes factor analysis provided moderate evidence for the null hypothesis in 8 of the 12 TEP peaks tested across sites, and weak evidence in the other peaks, suggesting that we were adequately powered to test our hypothesis. In line with our findings, TEPs following single-pulse TMS to premotor and parietal cortex are largely unaffected by anaesthetic doses of ketamine^20^, another NMDA receptor antagonist, suggesting that single-pulse TEPs are largely insensitive to changes in NMDA receptor-mediated neurotransmission. As NMDA receptors are dependent both on glutamatergic binding and depolarisation of the postsynaptic neuron, it is possible that a single TMS pulse is not sufficient to open NMDA receptors. Instead, paired-pulse TMS-EEG paradigms at intervals between 10–40 ms may be required to observe NMDA receptor-mediated neurotransmission^56^, similar to intracortical facilitation paradigms measured with MEPs in motor cortex^26^. Alternatively, early TEPs may reflect neurotransmission mediated by other ionotropic glutamate receptors, such as AMPA receptors, which requires further investigation. Finally, it is also possible that sensory-evoked activity following TMS may have masked changes in TMS-evoked cortical activity following NMDA receptor blockade, particularly for later peaks^10^.

### Effects of dextromethorphan on resting oscillations

Sub-anaesthetic doses of NMDA receptor antagonists, such as ketamine, have been reported to reduce power in delta, posterior theta and alpha oscillations, and increase frontal theta and gamma oscillations in human resting EEG^22^ and MEG^57^ recordings. We partially replicate these findings with dextromethorphan, showing reduced delta and theta oscillation power compared to placebo, however no changes in alpha or gamma oscillations. The reasons why dextromethorphan did not increase gamma oscillation power is unclear, although similar findings have been reported in animal models^58^. Our findings, however, add to the growing body of evidence demonstrating an important role for NMDA receptors in low frequency oscillations.

### Limitations of the study

A potential limitation of the current study is that the dose of dextromethorphan provided (120 mg) is lower than that required to produce hallucinations and cognitive impairment^59^, which are hallmarks of the effects of ketamine. However, we did observe modulation of low frequency resting oscillations similar to those observed with ketamine, and dextromethorphan at similar doses blocks paired-pulse and plasticity effects mediated by NMDA receptors in other TMS paradigms^26,27^, suggesting the dose here was adequate. Another potential limitation is that we only tested TEPs at one intensity. The effect of certain drugs can impact TEPs in a way which is dependent on stimulation intensity^60^. Furthermore, selecting an adequate stimulation intensity is important for optimising signal-to-noise ratios of early TEP peaks. While we did use anatomical MRI scans to individualise coil placement and observed differences in the scalp topography and source localisation of the early TEP peaks between stimulation sites, we did not check TEP amplitudes online to optimise stimulation intensity and minimise muscle artifact, a method which has recently been advocated to improve signal-to-noise in TMS-EEG recordings^11^. As a result, the early TEP peaks in this study are smaller than those observed by other groups stimulating similar regions^6^. Future studies assessing drug effects on TEPs should take into account a range of stimulation intensities and use online methods to ensure optimal stimulation intensities for eliciting early TEP peaks and avoiding artifacts. Finally, we only tested male participants in the current study to avoid the known effects of changing hormonal levels across the menstrual cycle on cortical excitability following TMS. Whether similar findings also hold in female participants requires further investigation.

## Conclusions

Our findings provide evidence that early TEP peaks following stimulation of prefrontal and parietal cortex in conscious human males are largely insensitive to changes in excitatory neurotransmission following NMDA receptor antagonism with dextromethorphan, at least at the dose tested. However, the early TEP peaks provide information specific to the site of stimulation, whereas late TEPs reflect activity less dependent on the stimulated sites. The role of NMDA receptor-mediated neurotransmission on modulating later peaks remains unclear, especially following parietal cortex stimulation. Future work using pharmacological agents targeting different excitatory and inhibitory receptor types is required to disentangle the physiological mechanisms contributing to early TEPs following TMS, and to test if these pharmacological effects are different when stimulating different cortical sites and following sensory control conditions.

## Supplementary information

Supplementary information.

## Data Availability

All data is available on request. Code is available at the following site: https://github.com/nigelrogasch/DXM_TMS-EEG_paper.
