# Supplementary material for: The effects of NMDA receptor blockade on TMS-evoked EEG potentials from prefrontal and parietal cortex
Source: Sci Rep. 2020 Feb 21;10:3168. doi: 10.1038/s41598-020-59911-6 (PMC7035341; doi:10.1038/s41598-020-59911-6)
Supplement: Supplementary file 1 — Supplementary information. [file 41598_2020_59911_MOESM1_ESM.pdf]

Supplementary materials

The effects of NMDA receptor blockade on TMS-evoked EEG potentials from prefrontal and parietal cortex

Nigel C. Rogasch[1,2,3], Carl Zipser[4], Ghazaleh Darmani[4], Tuomas P. Mutanen[5], Mana Biabani[1], Christoph Zrenner[4], Debora Desideri[4], Paolo Belardinelli[4], Florian Müller-Dahlhaus[4,6]*, Ulf Ziemann[4]*

*Equal contribution

1. Brain, Mind and Society Research Hub, School of Psychological Sciences and Turner Institute for Brain and Mental Health, Monash University, Melbourne, Australia
2. South Australian Health and Medical Research Institute (SAHMRI), Adelaide, Australia
3. Discipline of Psychiatry, Adelaide Medical School, University of Adelaide, Adelaide, Australia
4. Department of Neurology and Stroke, and Hertie Institute for Clinical Brain Research, Eberhard Karls University of Tübingen, Tübingen, Germany.
5. Centre for Cognitive Neuroimaging, Institute of Neuroscience and Psychology, University of Glasgow, Glasgow, United Kingdom
6. Department of Psychiatry and Psychotherapy, University Medical Center Mainz, Mainz, Germany

SUPPLEMENTARY METHODS

TMS-EEG cleaning pipeline two: For each site, data were epoched around the TMS pulse (-1500 to 1500 ms) and baseline corrected (-1000 to -10 ms). Data from -2 to 10 ms around the TMS pulse were removed and replaced by cubic interpolation prior to downsampling (1000 Hz), and then pre and post drug intake measurement time points concatenated so that independent component analysis (ICA) was applied equally across time. The data were then visually inspected, and epochs with excessive muscle or eye activity were removed, as were electrodes with excessive noise (e.g. from contact with the TMS coil). Data were then submitted to two rounds of FastICA [1]. In the first round, independent components representing TMS-evoked muscle or large decay artifacts were detected using the TESA compselect function (default settings) and manually checked before being removed [2,3]. Data were then bandpass (1-100 Hz) and bandstop (48-52 Hz) filtered using a zero-phase butterworth filter (order = 4) prior to the second round of FastICA, in which components representing blinks, eye movement, muscle activity or electrode noise were detected with TESA and manually removed. Finally, missing electrodes were replaced using spherical interpolation before data were re-referenced to the average of all electrodes, and separated back into individual time points. See table S2 for details on number of trials, channels and components removed.

Table S1: *Total trials, number of channels removed, and number of components removed during cleaning of TEPs using pipeline one.*

Site, condition, time	Total trials	Channels removed	Components removed (ICA1)	Components removed (ICA2)	Components removed (SSP-SIR)
PFC, DXM, Pre	135.9 [124-145]	0.8 [0-1]	1.9 [1-5]	10.4 [4-23]	0.4 [0-2]
PFC, DXM, Post	130.0 [95-144]	0.7 [0-1]	2.3 [1-5]	10.6 [3-22]	0.6 [0-4]
PFC, PBO, Pre	137.8 [128-145]	0.8 [0-1]	2.0 [1-4]	12.6 [3-27]	0.4 [0-2]
PFC, PBO, Post	136.6 [113-144]	0.8 [0-1]	1.9 [1-4]	9.9 [2-19]	0.4 [0-2]
PAR, DXM, Pre	133.5 [115-142]	0.9 [0-1]	2.7 [1-4]	11.3 [1-29]	0.7 [0-4]
PAR, DXM, Post	128.9 [92-145]	0.8 [0-1]	2.5 [0-4]	14.9 [4-26]	0.8 [0-3]
PAR, PBO, Pre	132.9 [111-144]	0.8 [0-1]	2.6 [1-5]	12.8 [3-28]	0.6 [0-1]
PAR, PBO, Post	139.6 [134-146]	1.1 [0-2]	2.7 [2-5]	9.1 [3-21]	1.5 [0-4]

NB: *Data are mean [range]. DXM, dextromethorphan; ICA, independent component analysis; PAR, parietal cortex; PBO, placebo; PFC, prefrontal cortex; SSP-SIR, signal-space-projection source-informed reconstruction; TEP, TMS-evoked potential.*

Table S2: *Total trials, number of channels removed, and number of components removed during cleaning of TEPs using pipeline two.*

Site, condition, time	Total trials	Channels removed	Components removed (ICA1)	Components removed (ICA2)
PFC, DXM, Pre	134.6 [128-142]	1.5 [0-5]	0.9 [0-3]	30.7 [24-38]
PFC, DXM, Post	132.0 [92-141]			
PFC, PBO, Pre	135.5 [129-140]	1.8 [0-5]	0.9 [0-3]	29.7 [22-37]
PFC, PBO, Post	133.2 [126-138]			
PAR, DXM, Pre	134.1 [116-142]	1.3 [0-3]	2.1 [0-5]	33.9 [24-44]
PAR, DXM, Post	132.5 [92-140]			
PAR, PBO, Pre	131.1 [97-140]	1.6 [0-5]	1.6 [0-4]	32.1 [15-43]
PAR, PBO, Post	135.2 [128-141]			

NB: *Data are mean [range]. Missing values indicate when data were concatenated and cleaning was applied equally between pre and post time points. DXM, dextromethorphan; ICA, independent component analysis; PAR, parietal cortex; PBO, placebo; PFC, prefrontal cortex; TEP, TMS-evoked potential.*

Table S3: *Total segments, number of channels removed, and number of components removed during cleaning of resting EEG.*

State, condition, time	Total segments	Channels removed	Components removed
Open, DXM, Pre	124.4 [103-165]	0 [0-0]	21.8 [13-31]
Open, DXM, Post	124.1 [114-139]		
Open, PBO, Pre	126.1 [85-162]	0 [0-0]	21.3 [11-29]
Open, PBO, Post	122.5 [82-165]		
Closed, DXM, Pre	122.1 [110-147]		
Closed, DXM, Post	134.6 [103-197]		
Closed, PBO, Pre	121.8 [75-172]		
Closed, PBO, Post	125.8 [88-168]		

NB: *Data are mean [range]. Missing values indicate when data were concatenated and cleaning was applied equally between pre and post time points and eyes open and eyes closed states. DXM, dextromethorphan; EEG, electroencephalography; PBO, placebo.*

SUPPLEMENTARY RESULTS

Effect of dextromethorphan on blood pressure

There was a main effect of time on diastolic blood pressure ($F_{1,13}$=7.9, p=0.006, RM-ANOVA), with pressure increasing across the experiment, however we could not detect any main effect of condition ($F_{1,13}$=0.2, p=0.71), or time $\times$ condition interaction ($F_{1,13}$=0.4, p=0.66). We could not detect any main effects of time ($F_{1,13}$=2.4, p=0.13) or condition ($F_{1,13}$=0.0, p=0.90), or a time $\times$ condition interaction ($F_{1,13}$=1.1, p=0.34) on systolic pressure, suggesting dextromethorphan and placebo did not have differential effects on blood pressure.

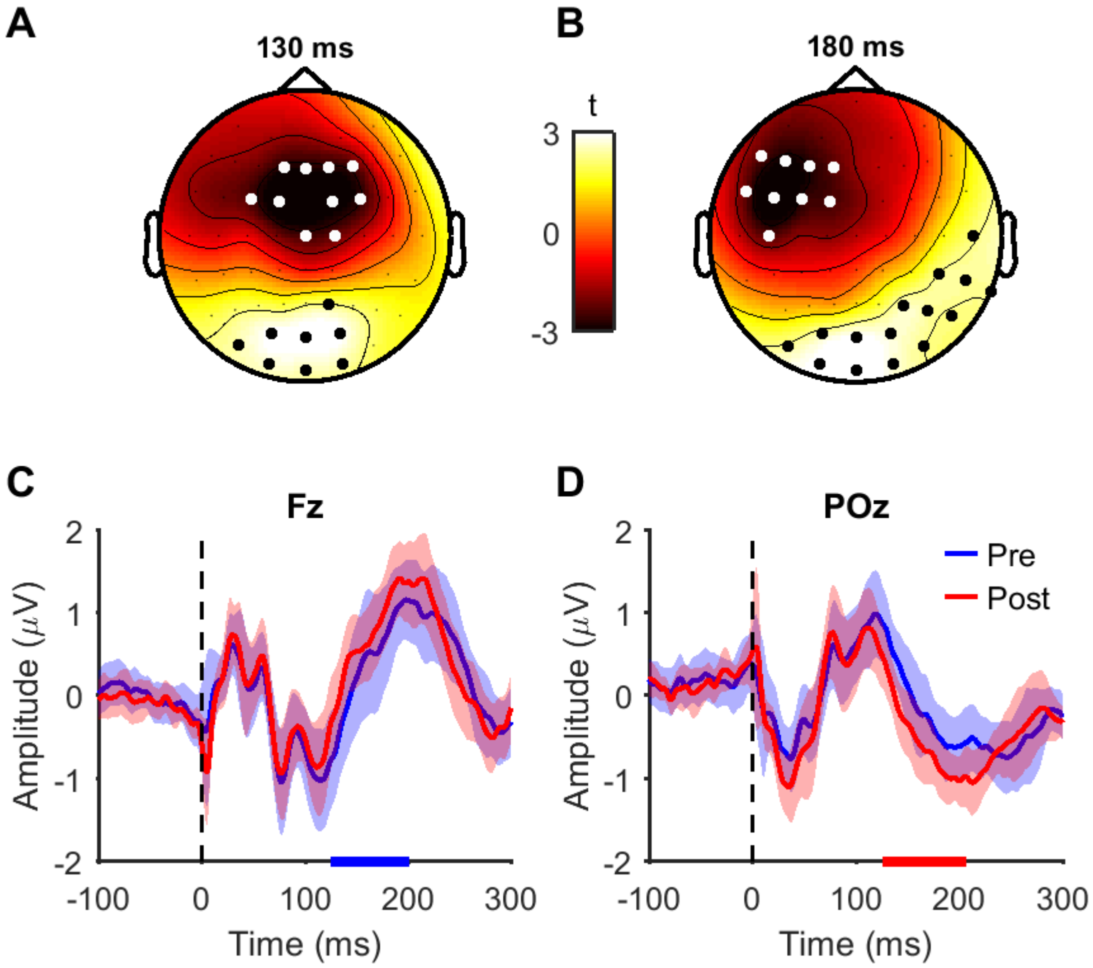

Figure S1: Changes in TEP amplitudes following dextromethorphan after parietal cortex stimulation. *A-B) Topoplots displaying t-statistics at two time points during significant clusters indicating differences in TEP amplitudes pre and post dextromethorphan administration (positive cluster, p=0.006, 126-207 ms, black dots; negative cluster, p=0.0132, 125-201 ms, white dots). C-D) Plots from single electrodes in negative (C) and positive (D) clusters pre and post dextromethorphan administration. Shaded bars represent 95% confidence intervals. Solid bars on x axis indicate timing of significant negative (blue) and positive (red) clusters. Dashed black line indicates timing of the TMS pulse.*

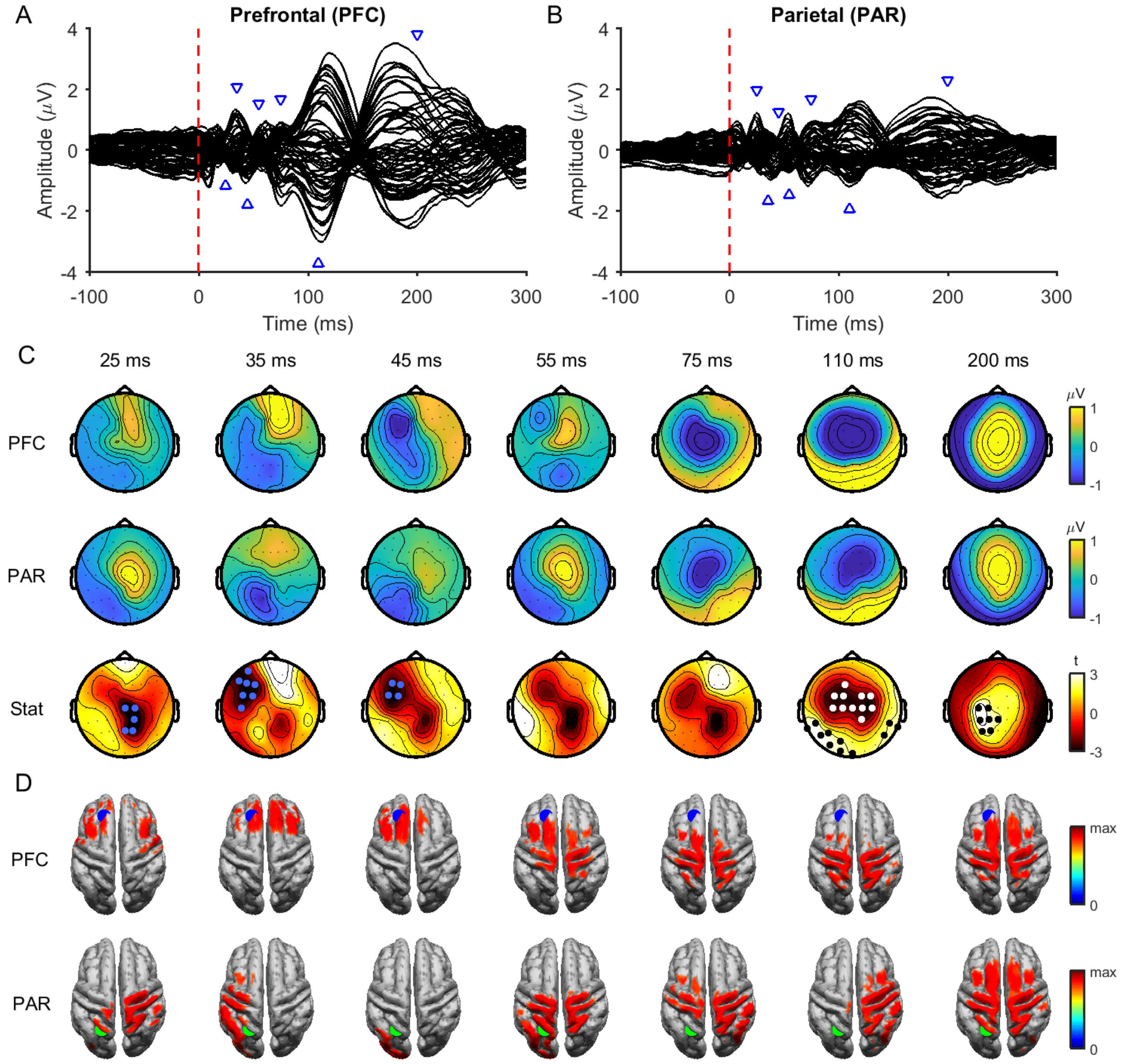

Figure S2: Comparison of baseline TEPs between stimulation sites using TMS-EEG cleaning pipeline two. *Butterfly plots of grand average TEPs across all individuals following prefrontal (PFC; A) and parietal (PAR; B) cortex stimulation at baseline (averaged across conditions). The red dashed line represents the timing of the TMS pulse and the blue triangles the latencies plotted in C and D. C) Topoplots showing the grand average amplitude of TEPs at different time points following PFC (top row), and PAR stimulation (middle row). The bottom row shows t-statistics comparing the amplitude of PFC and PAR stimulation. White and black dots*

indicate significant negative and positive clusters ($p<0.05$; cluster-based permutation tests on 15-250 ms). Blue dots indicate significant clusters over shorter time windows (15-30 ms; 31-45 ms). D) Minimum-norm estimate source maps averaged across participants showing peak activity at each time point in C following PFC (top row) and PAR (bottom row) stimulation. Activity has been thresholded to 85% of maximum activity at each time point. The blue dot represents the target for PFC stimulation and the green dot the target for PAR stimulation.

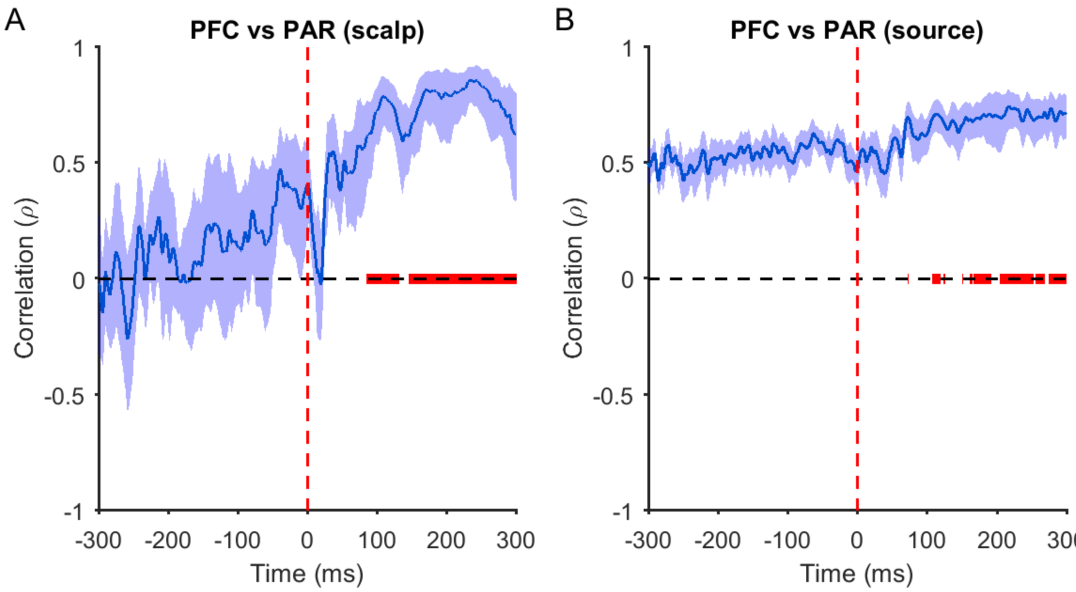

Figure S3: Spatial correlations between prefrontal (PFC) and parietal (PAR) TEPs following TMS-EEG cleaning pipeline two. *Spearman correlations comparing the relationship between PFC and PAR TEPs at the scalp (A) and source (B, using MNE) level for each time point. The thick blue line represents the mean rho values across individuals, and the shaded bars the 95% confidence intervals. The thick red line indicates post stimulation time points where correlations are greater than at equivalent pre stimulation time points (p<0.05; Mann-Whitney U test). Note that rho values were converted to z for statistics, then back to rho for plotting.*

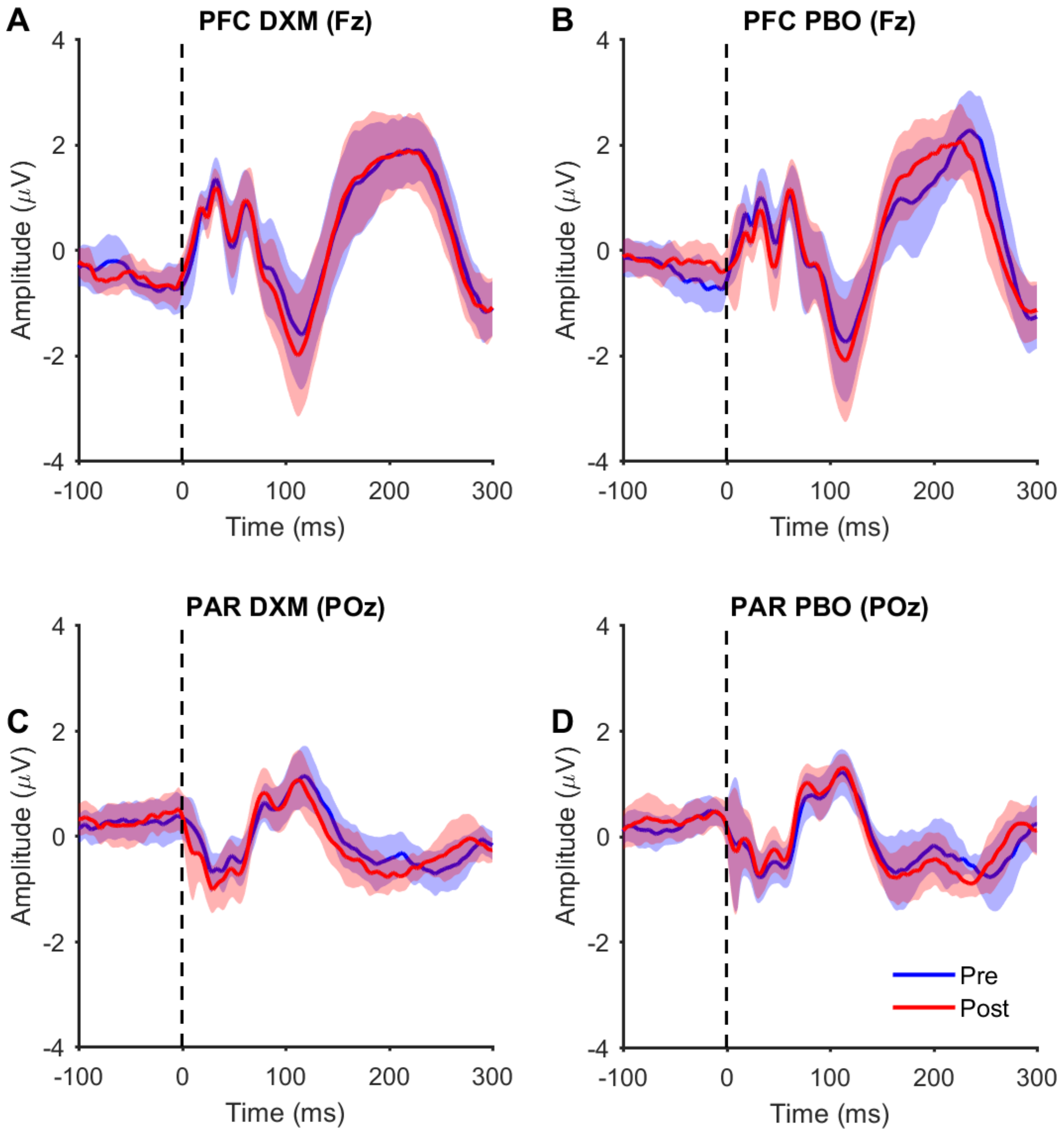

Figure S4: TEPs from single electrodes following dextromethorphan (DXM) and placebo (PBO) using TMS-EEG cleaning pipeline two. *A-B) TEPs measured from the Fz electrode following prefrontal cortex (PFC) stimulation pre and post dextromethorphan (DXM) and placebo (PBO) administration. C-D) TEPs measured from the POz electrode following parietal (PAR) cortex stimulation pre and post dextromethorphan and placebo administration. Thick coloured lines represent the group mean and shaded colour lines represent 95% confidence intervals.*

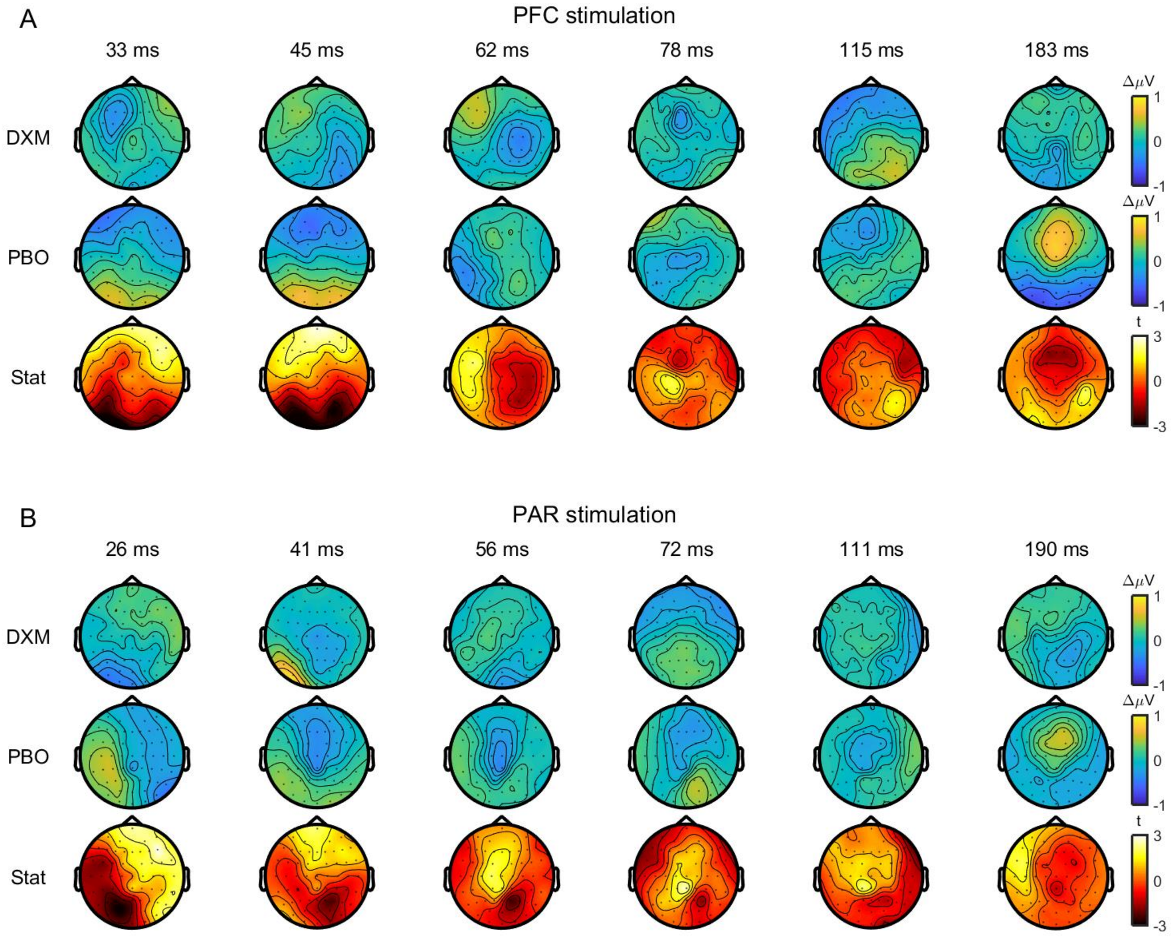

Figure S5: Comparison of changes in TEPs following dextromethorphan (DXM) and placebo (PBO) using TMS-EEG cleaning pipeline two. *Topoplots showing changes in TEP amplitude at peak latencies following prefrontal (PFC; A) and parietal (PAR; B) cortex stimulation after dextromethorphan (top row) and placebo (middle row). Topoplots showing t-statistics (within-subject t-tests) comparing TEP changes between dextromethorphan and placebo are shown on the bottom row. No differences were observed between conditions (cluster-based permutation tests).*

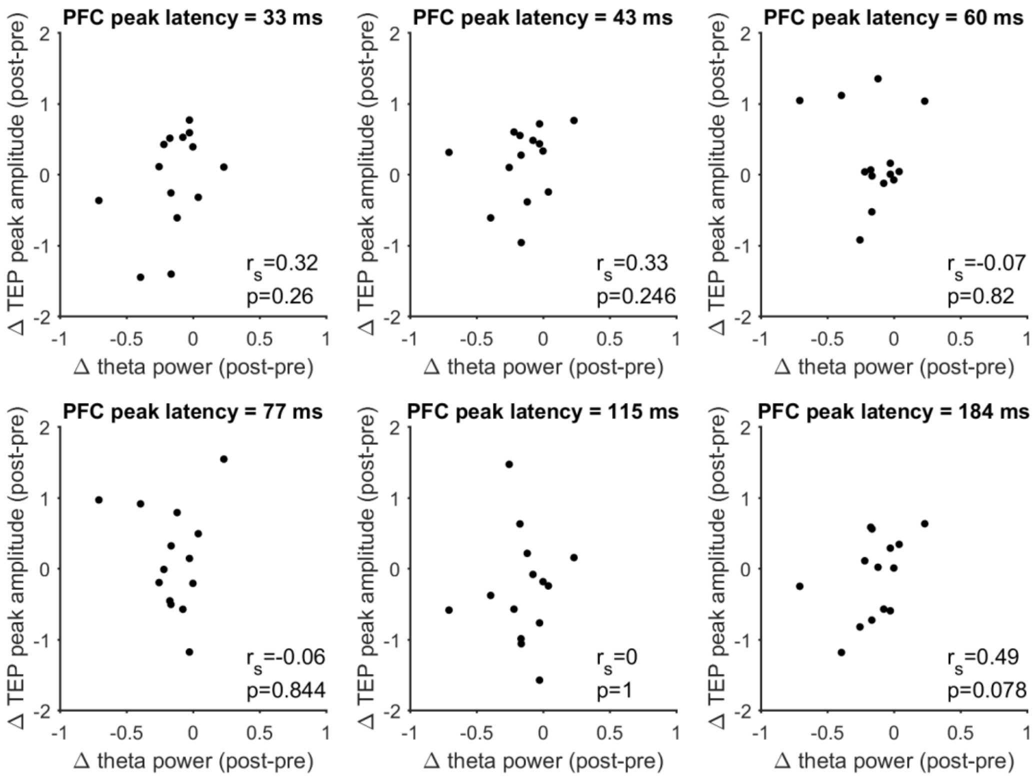

Figure S6: Correlation between changes in resting theta oscillatory power and changes in prefrontal cortex (PFC) TEP peak amplitudes following dextromethorphan.

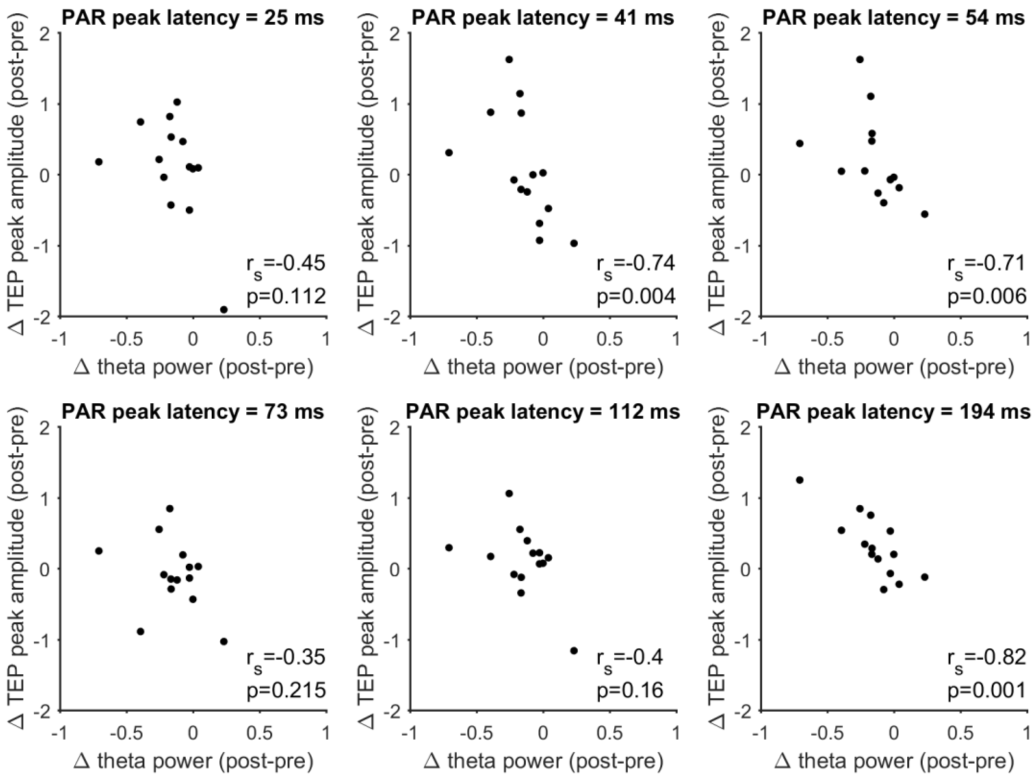

Figure S7: Correlation between changes in resting theta oscillatory power and changes in parietal cortex (PAR) TEP peak amplitudes following dextromethorphan.

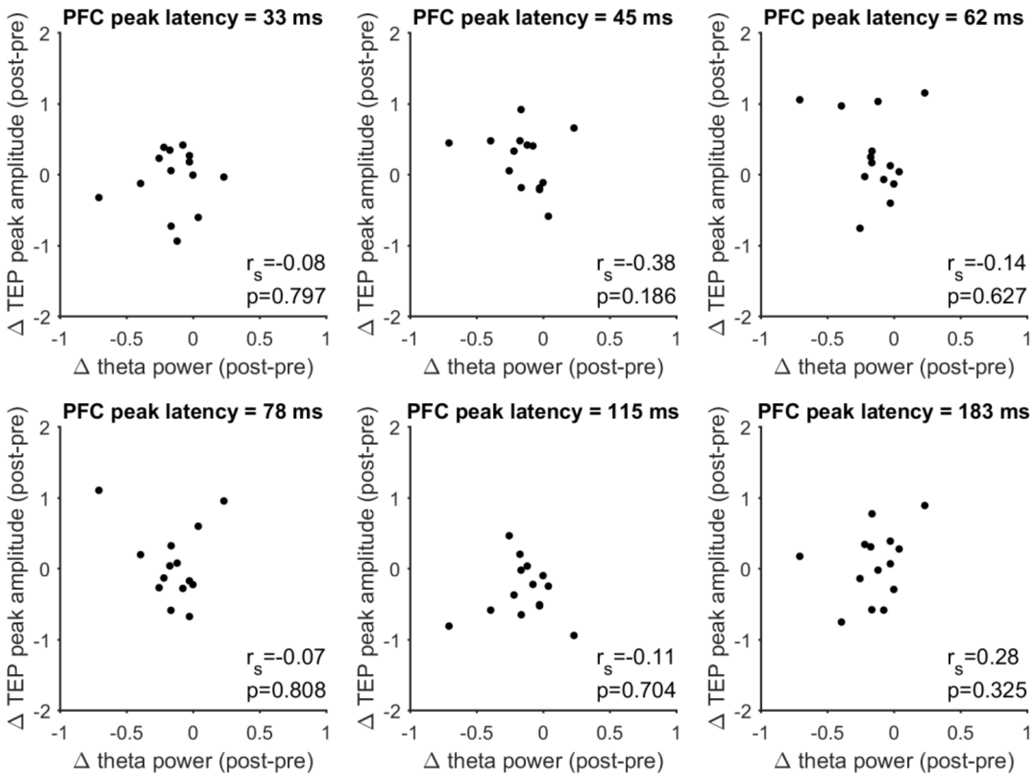

Figure S8: Correlation between changes in resting theta oscillatory power and changes in prefrontal cortex (PFC) TEP peak amplitudes following dextromethorphan using TMS-EEG cleaning pipeline two.

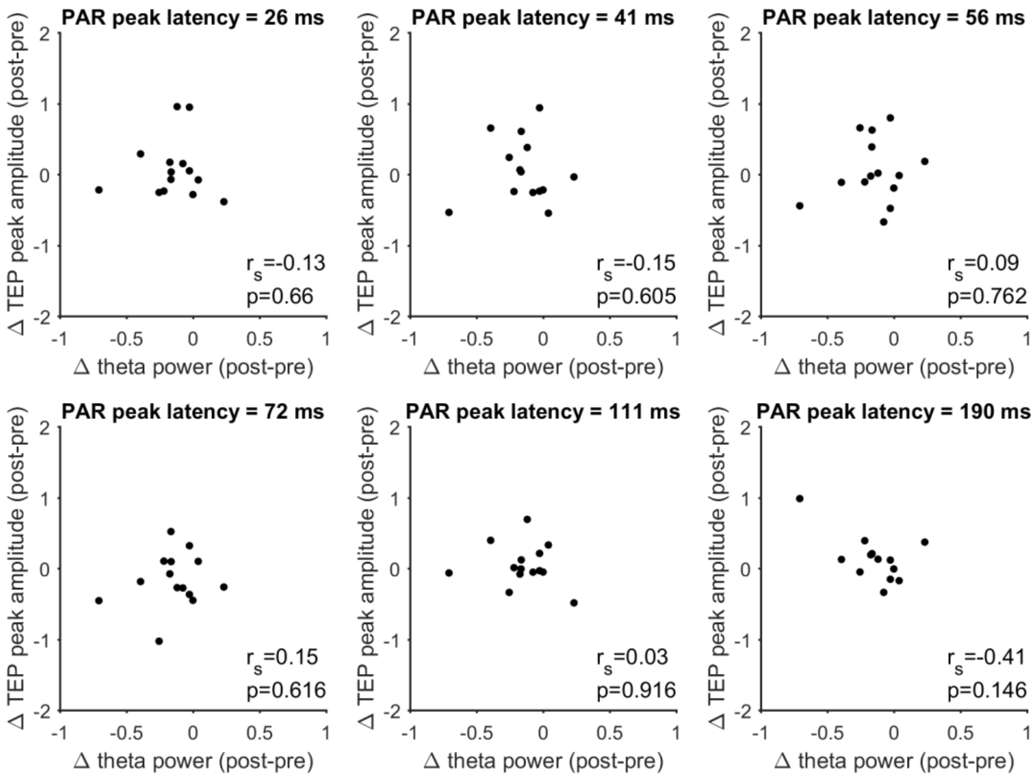

Figure S9: Correlation between changes in resting theta oscillatory power and changes in parietal cortex (PAR) TEP peak amplitudes following dextromethorphan using TMS-EEG cleaning pipeline two.

Table S4: *Distance from TMS target sites to best-fitting dipoles at baseline using pipeline two.*

	**Distance from target (mm)**	**Distance from non-target (mm)**	**Goodness of fit (GoF)**	**p-value**
PFC (15-45 ms)	**54 [21-100]**	70 [41-92]	0.89 [0.80-0.96]	0.046
PFC (95-125 ms)	94 [58-134]	**52 [15-89]**	0.86 [0.65-0.96]	$2.16x10^{-4}$
PFC (175-205 ms)	80 [41-128]	**52 [33-90]**	0.85 [0.68-0.964]	$8.65x10^{-4}$
PAR (15-45 ms)	**48 [20-84]**	87 [43-139]	0.89 [0.65-0.95]	$2.59x10^{-4}$
PAR (95-125 ms)	**55 [24-88]**	94 [78-114]	0.89 [0.74-0.98]	$3.91x10^{-5}$
PAR (175-205 ms)	**46 [9-70]**	80 [44-131]	0.83 [0.64-0.98]	$8.55x10^{-5}$

NB: *Values in column 1-3 represent the mean [range]. Bold numbers indicate which site was closest to the best fitting dipole (target vs. non-target; p<0.05, Mann-Whitney U test). PFC, prefrontal cortex; PAR, parietal cortex.*

Table S5: *Bayes factors comparing the change in TEP peak amplitude following dextromethorphan (DXM) vs. placebo (PBO) using pipeline two.*

	**DXM vs PBO**	
**TEP peaks**	**PFC (BF_{01})**	**PAR (BF_{01})**
33, 25	0.9	1.7
43, 41	0.9	**3.5**
60, 54	**3.7**	2.1
77, 73	**3.4**	1.2
115, 112	**3.6**	2.8
184, 194	2.8	2.8

NB: *Values in column one represent the mean TEP peak latency for prefrontal cortex (PFC) and parietal cortex (PAR) stimulation respectively. Bold numbers indicate moderate evidence for no difference between conditions.*

REFERENCES

1. Hyvärinen, A. & Oja, E. Independent component analysis: algorithms and applications. *Neural Netw.* **13**, 411–430 (2000).
2. Rogasch, N. C. *et al.* Removing artefacts from TMS-EEG recordings using independent component analysis: importance for assessing prefrontal and motor cortex network properties. *Neuroimage* **101**, 425–439 (2014).
3. Rogasch, N. C. *et al.* Analysing concurrent transcranial magnetic stimulation and electroencephalographic data: A review and introduction to the open-source TESA software. *Neuroimage* **147**, 934–951 (2017).
